# Shallow stability and parameter sensitivity analysis of soil slope with frame protection under rainfall seepage

**DOI:** 10.1038/s41598-021-99181-4

**Published:** 2021-10-04

**Authors:** Jifeng Lian, Jiujiang Wu

**Affiliations:** 1 School of Emergency Science, Xihua University, Chengdu, 610039 Sichuan China; 2 Shock and Vibration of Engineering Materials and Structures Key Laboratory of Sichuan Province, Southwest University of Science and Technology, Mianyang, 621010 Sichuan China; 3 College of Civil Engineering and Architecture, Wenzhou University, Wenzhou, 325035 Zhejiang China

**Keywords:** Civil engineering, Natural hazards

## Abstract

Frame protection is a commonly used solution to maintain the shallow stability of soil slope under rainfall seepage. Currently, the frame structure's design is empirical, and its theoretical analysis method considering the influence of seepage is scarce. Based on the instability model of the infinite slope, the shallow stability calculation model of soil slope under the rectangular frame protection is established in this paper. The calculation results show that it is beneficial to maintain the shallow slope stability by reducing the skeleton spacing and increasing the cross-sectional size of the frame structure. Also, geometric parameters' sensitivity analysis of the frame structure is carried out based on the orthogonal experimental design methods. Therein, an optimal scheme evaluation function was constructed to balance the relationship between the safety factor and the construction material consumption. The calculation model and results included in this paper can guide the design of the rectangular frame protection to soil slope under rainfall seepage.

## Introduction

Rainfall is the most critical environmental factor that induces soil slope instability^1^. The survey shows that more than 90% of the roadbed soil slope disaster appears in the rainy season, of which the shallow slide is the most typical^2^. The shallow instability of the roadbed slope caused by rainfall is different from a general landslide. It has the characteristics of shallow sliding depth and small scale, high frequency, wide distribution, strong suddenness, and easy formation of chain effects^3^, which also brought serious hazards to railways or highways.

**Figure 1:**
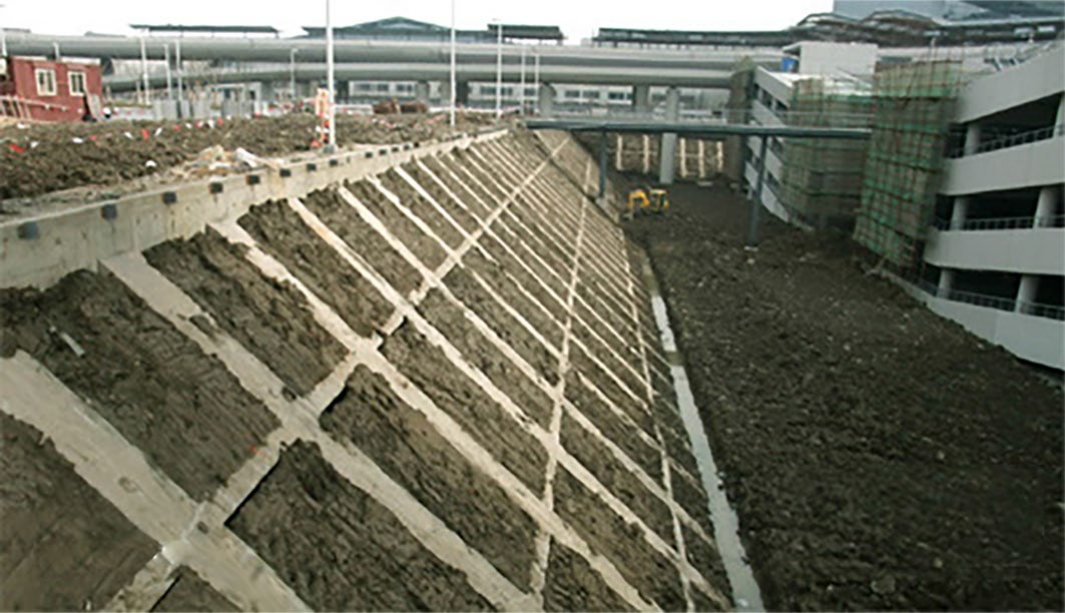
Frame protection of soil slope.

In 2005, the loess (silt) embankment of the Baozhong Railway in China encountered heavy rainfall, which caused multiple shallow slope slides with a sliding depth of 0.5 to 1.2 m, resulting in the limitation of train speed to prevent the triggering of derail^4^. On August 21, 2012, the sudden heavy rain near the Guangde station of the Xuan-Hang line caused an approximately 50 m^2^ shallow slope slide with 0.6–0.7 m depth to a high embankment. To ensure traffic safety, public works of the section organized employees to temporarily take the rain to rescue and strengthen the embankment^5^. Concerning the highway field, shallow slope failure can be frequently found in Southern California of the United States during the rainy season, and there is no noticeable improvement effect to the embankment after refilling^6^. After the Algeria East–West Expressway was completed and opened to traffic, various types of disasters appeared on the embankment's side slope after three rainy seasons, among which the shallow failure accounted for more than 90%^7^. At present, in order to prevent the shallow instability and destruction of the subgrade soil slope caused by heavy rainfall, the slope protection design adopts the frame structure as a typical type of slope protection, as illustrated in Fig. 1.

The frame structure can be various^8^, for example, rectangular sash, arched sash, diamond sash, etc. Generally, the material used for the frame protection structure is mortar masonry rubble or plain concrete, and the frame structure used on weathered rock slopes is composed of steel frame and concrete, and anchor rod is commonly punched at the frame nodes. The bearing mechanism of the frame structure with anchor rods is relatively complicated. Currently, the engineering design of the frame protection structure is empirical. According to the engineer's experience, the clear distance of the skeleton structure is set to 2–4 m, and the section size is between 0.2 and 0.4 m^9,10^. Meanwhile, although the interaction between the frame structure and the shallow sliding slope body was analyzed^6,11,12^, the impact of rainfall seepage was not considered.

Generally speaking, the frame protection structure of the subgrade soil slope is still based on empirical design. The theoretical analysis method for the protection structure design considering the influence of rainfall seepage is scarce, especially for the influence of the frame structure's geometric parameters on the shallow slope stability. Based on the instability model of the infinite slope, the rectangular frame structure is used as the analysis object in this paper. The shallow slope stability calculation model under the rectangular frame protection is established considering the stabilization effect of the frame structure. Based on the orthogonal experimental design methods, geometric parameters' sensitivity analysis of the frame structure is carried out. The calculation model and results included in this paper can guide the design of the rectangular frame protection to soil slope under rainfall seepage.

## Conventional infinite-slope stability analysis

**Figure 2:**
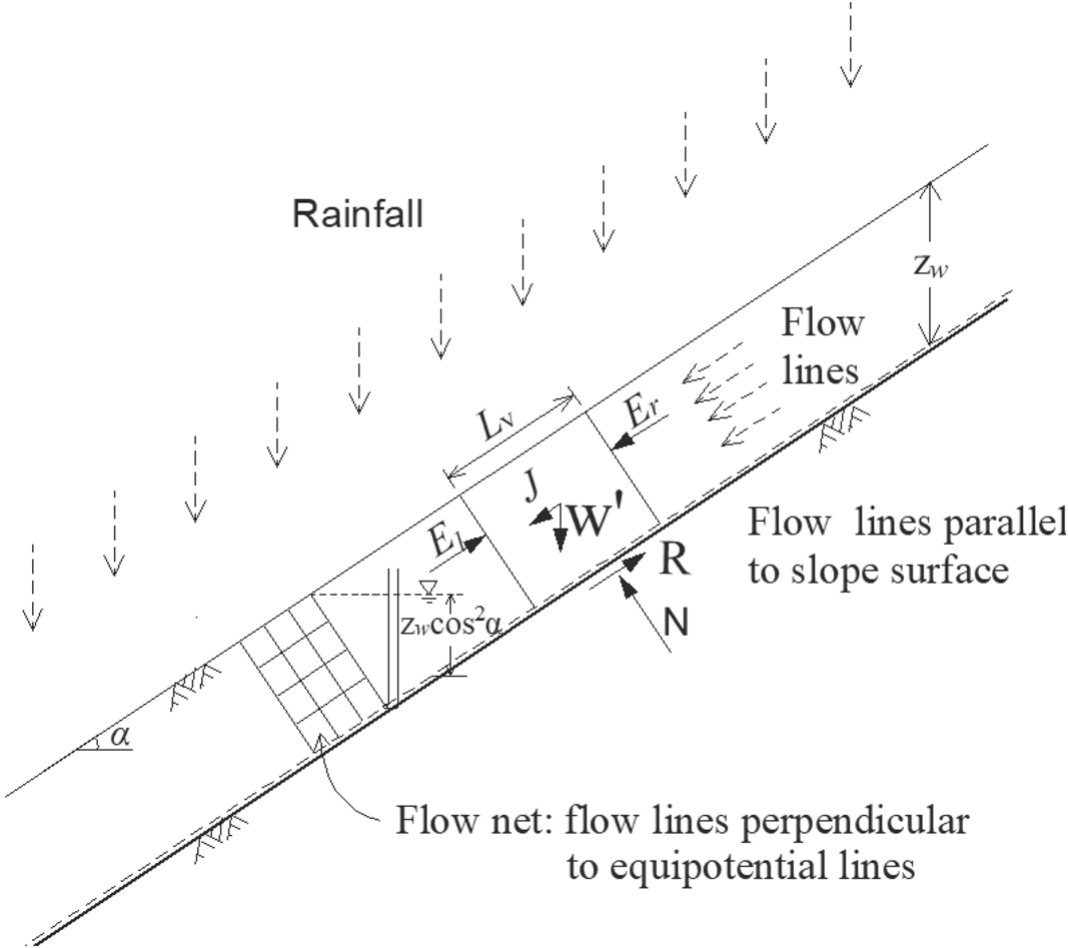
Shallow translational slide.

When heavy rains infiltrate into the upper layers of soil slope and saturate the soil, and then rainwater enters the slope producing seepage parallel to the surface by an impervious layer at some depth, as shown in Fig. 2, slope failure begins to take place^6,13–16^. For the infinite slope with incline angle *α* and the water infiltration depth z_*w*_, and any length *L*_v_ of soil block, *E*_l_ and *E*_r_ are earth pressures at the two end of the block, parallel to the slope surface, opposite force and equal in magnitude, and they are not considered under an infinite slope condition.

The water pressure *u* at the vertical depth z_*w*_ is:

1 $$u = \gamma_{w} z_{w} \cos^{2} \alpha$$

where *γ*_w_ is the unit weight of water.

The seepage force *J* acting on the block is:

2 $$J = L_{v} \gamma_{{\text{w}}} iz_{w} = L_{v} \gamma_{{\text{w}}} z_{w} \sin \alpha \cos \alpha$$

where *i* is the hydraulic gradient and equal to $\sin \alpha$.

The effective vertical force $W^{\prime}$ of the block is:

3 $$W^{\prime} = \gamma^{\prime}z_{w} L_{v} \cos \alpha$$

where $\gamma^{\prime}$ is the buyout unit weight of the soil.

The effective vertical force *Wʹ* is resolved into the effective normal force, *Nʹ*, and the driving force, *Sʹ*. The effective normal force, *Nʹ*, is then calculated as:

4 $$N^{\prime} = W^{\prime}\cos \alpha$$

For the stability of the block with depth *z*_*w*_ and length *L*_v_, the available resistance force of the block according to the Coulomb formula is:

$$R = (c^{\prime} + \gamma^{\prime}z_{w} \cos \alpha \tan \varphi^{\prime})L_{v}$$

where *cʹ* is the effective cohesion, and *φ*ʹ is the effective inner frictional angle.

And then the total driving force *S* of the block is:

6 $$S = J + W^{\prime}\sin \alpha$$

So if we define the factor of safety *F*_s_ as the ratio of the total resistance force to the driving force of the block, the *F*_si_ will be given by

$$F_{{{\text{si}}}} = \frac{R}{S} = \frac{{(c^{\prime} + \gamma^{\prime}z_{{\text{w}}} \cos^{2} \alpha \tan \varphi^{\prime})L_{{\text{v}}} }}{{(\gamma_{{{\text{sat}}}} z_{{\text{w}}} \cos \alpha \sin \alpha )L_{{\text{v}}} }}$$

In fact, the method is based on the shear in the translational plane; whether or not it reaches the shear strength, it cannot consider the resistant force of upper and lower of the sliding, so the result of the method will be safer than the actual state^17^. It should be noted that the method will be more reasonable while the length *L*_v_ of the sliding block is larger than the depth z_*w*_.

## The mechanical analysis of the structure and soil mass

**Figure 3:**
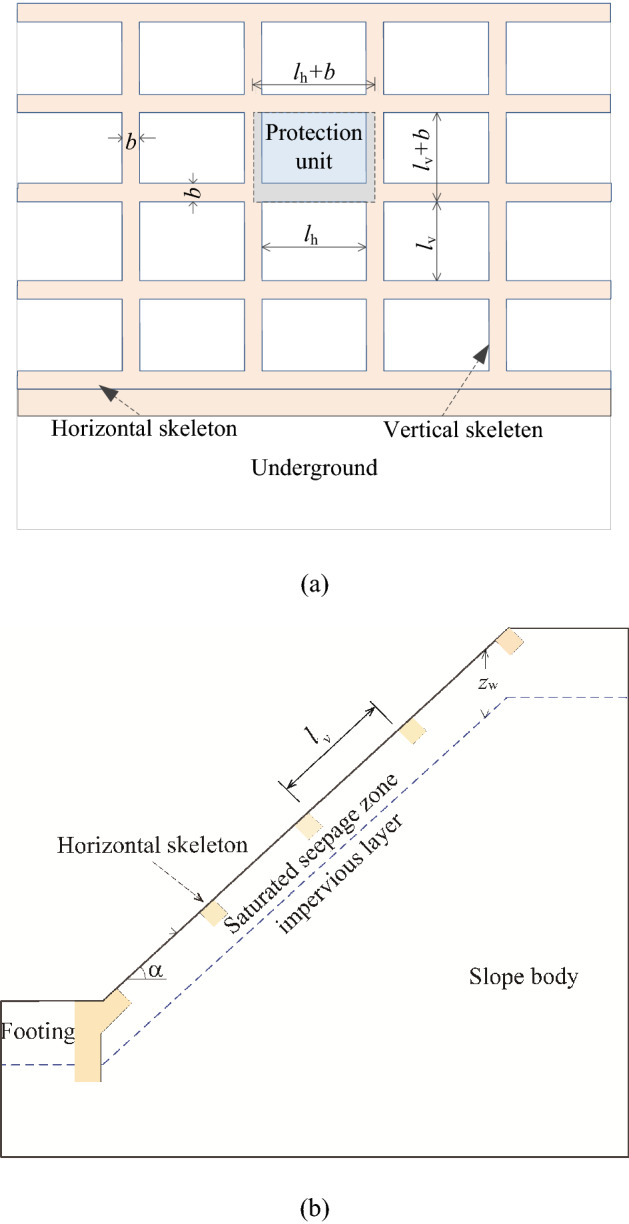
Subgrade slope protection with concrete skeleton: **(a)** plan view; **(b)** elevation view.

The rectangular frame protection structure consists of vertical skeletons, horizontal skeletons, and footing. According to the practical design requirements, the footing will be embedded in the deep hard soil layer to improve stability. Vertical skeletons and horizontal skeletons have the same width *b* and thickness *h*. The horizontal and vertical clear distances between the skeletons are *l*_h_ and *l*_v_, as shown in Fig. 3. Skeleton structure divides the sliding force of effective gravity of the surficial layers into small parts. Under the rectangular skeleton structure protection, the surficial failure mode of soil slope is assumed to be translational sliding.

It should be noted that the assumptions of the calculation model are based on the theory of infinite slopes and can be described as follows:

(1) The shallow instability mode of subgrade slope is classified as the translational slide failure mode;

(2) Surficial seepage flows are parallel to the slope surface;

(3) The frame structure is supported by the footing to keep itself stable.

**Figure 4:**
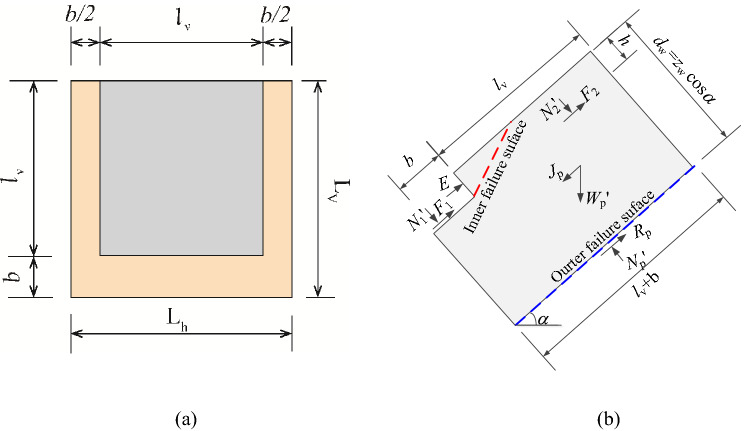
the protection cell and force: **(a)** the protection cell; **(b)** force on the isolated soil element.

The resistant effects of skeleton structure on upper layer soil of slope mainly consist of contributions from retaining force, *E*, which is opposite to earth pressure produced by the horizontal skeleton structure, and from bottom frictional resistance induced by the skeletons, *F*, existing at the contact surface between skeleton structure and soil. The shear strength of soil mass under the skeleton structure has also been enhanced due to normal stress increasing induced by the skeleton structure's self-weight, of which component parallel to the slope surface can be equilibrated by the foundation counterforce at the slope toe. It is assumed that the side friction between horizontal skeleton structure and soil mass is equal to zero because of no displacement producing at the direction perpendicular to the slope surface under infinite slope conditions. Besides, the side friction of the vertical skeleton can also be ignored due to its weak effect on soil mass compared to the retaining force *E* and bottom frictional forces *F*_1_, *F*_2_. The skeleton protection unit with length *L*_v_ and width *L*_h_ is shown in Fig. 4a. It can be seen that *L*_v_ and *l*_v_ have different meanings and *L*_v_ = *l*_v_ + *b*.

## Stability analysis considering the anti-sliding effect of frame structure

Usually, the net distance *l*_v_ and *l*_h_ is 2–4 m, and the skeleton section width *b* and thickness is 0.3–0.5 m^9,10^. When the bottom of the soil cell is sliding along the outer failure surface, the second failure surface, i.e., the inner failure surface, will be generated due to the retaining force *E* of the horizontal skeleton, as shown in Fig. 4b. By using the limit equilibrium condition, the limit resistance of the soil cell sliding $R_{{\text{p}}} = \left( {N_{{\text{p}}}^{\prime } \tan \varphi^{\prime} + c^{\prime}L_{{\text{v}}} } \right)/F_{{\text{s}}}$, the factor of safety *F*_s_ can be solved as follow:

8 $$F_{{\text{s}}} = \frac{{R_{{\text{p}}} }}{{S_{{\text{p}}} }} = \frac{{N_{{\text{p}}}^{\prime } \tan \varphi^{\prime} + c^{\prime}L_{{\text{v}}} }}{{W_{{\text{p}}}^{\prime } \sin \alpha + J_{{\text{p}}} - F_{1} - F_{2} - E}}$$

where $S_{{\text{p}}}$ is the soil cell sliding force; $N_{{\text{p}}}^{\prime }$ is the effective normal pressure at the bottom of the unit soil; $W_{{\text{p}}}^{\prime }$ is the effective gravity of the soil cell; *J*_p_ is the permeability of the soil cell under downslope seepage; *F*_1_ and *F*_2_ are the frictional forces of the horizontal frame and the vertical frame against the sliding body, respectively; *E* is the soil resistance; the variables listed above can be determined by Eq. (9).

9 $$\left\{ \begin{gathered} F_{1} + F_{2} = W^{\prime}_{{\text{c}}} \cos \alpha \tan \delta \hfill \\ W_{{\text{p}}}^{\prime } = \frac{{\gamma^{\prime}d_{w} (L_{{\text{v}}} L_{{\text{h}}} - V_{{\text{c}}} )}}{{l_{{\text{h}}} + b}} \hfill \\ J_{{\text{p}}} = \frac{{\gamma_{{\text{w}}} d_{w} (L_{{\text{v}}} L_{{\text{h}}} - V_{{\text{c}}} )i}}{{l_{{\text{h}}} + b}} \hfill \\ E = E_{{\text{p}}}^{\prime } + J_{{\text{u}}} \hfill \\ N_{{\text{p}}}^{\prime } = W_{{\text{p}}}^{\prime } \cos \alpha \hfill \\ \end{gathered} \right.$$

where: $W_{{\text{c}}}^{\prime }$ is the effective gravity of the unit frame, and can be defined as $\gamma_{{\text{c}}}^{\prime } V_{{\text{c}}}$; *V*_c_ is the volume of the unit frame, and can be calculated by $\left[ {l_{{\text{v}}} + (l_{{\text{h}}} + b)} \right]bh$; *J*_u_ is the infiltration force of the triangular wedge when the passive earth pressure fails and can be described as $hl_{{\text{p}}} \gamma_{{\text{w}}} i$; *E*_p_' is the effective passive earth pressure. According to Coulomb's passive earth pressure theory^17^, the *E*_p_' is given by

10 $$E_{{\text{p}}}^{\prime } = \frac{1}{2}K_{{\text{p}}} \gamma^{\prime}(h\cos \alpha )^{2}$$

When there is no frame protection, *N*' = *N*_p_'*, W*' = *W*_p_'*, F*_1_, *F*_2_, and *E* are all equal to 0, and Eq. (7) can degenerate into the safety factor *F*_si_ definition of infinite slope stability^6,13^.

## Orthogonal experimental design

In this paper, the iso-level orthogonal table is denoted by the *L*_n_(*r*^m^). Among them, *L* is the code of the orthogonal table; *n* is the number of rows in the orthogonal table, i.e., the number of experiments that should be done; *m* is the number of columns in the orthogonal table, i.e., the maximum number of influencing factors that can be arranged^18^. Orthogonal design tables provide corresponding tables according to different factors and levels, such as orthogonal tables L_4_(2^3^), *L*_8_(2^7^), *L*_9_(3^4^), etc.

In the statistical analysis of the calculation results, let A, B, … be different factors: A_i_ is the i-th level of factor A (i = 1, 2, …, *r*); *r* is the level numbers of each factor; X_ij_ is the value of the i-th level of j (i = 1, 2,…, *r*; j = A, B, …). Calculate the safety factor *F*s_ij_ of the i-th level of factor j under *X*_ij_. Meanwhile, *n*_1_ times of tests are carried out under X_ij_, and the calculated safety factors are represented by *F*_sijk_ (k = 1, 2, …, *n*_1_). The statistical parameter *K*_ij_ of factor j at the level of i is expressed by Eq. (11).

11 $$K_{ij} = \sum\limits_{k = 1}^{{n_{1} }} {F_{sijk} }$$

where *n*_1_ is the number of calculations that factor j participates in at the level i, which is determined by the selected orthogonal table; the range value R is calculated according to the statistical parameters *K*_ij_ of each factor, namely

12 $$R_{j} = \max \left\{ {K_{1j} ,K_{2j} , \cdots ,K_{rj} } \right\} - \min \left\{ {K_{1j} ,K_{2j} , \cdots ,K_{rj} } \right\}$$

The magnitude of the range value *R* reflects the influence degree of the factor level change on the test result. The larger the range value, the greater the influence of the factor level change on the test result; that is, the higher the significance of the factor.

## Case study

Take the slope of a compacted soil roadbed in Southern California^6^ as a case history. According to the investigation, a large area of landslide occurred in Southern California after heavy rainfall, the sliding depth was mostly 0.5–1.0 m, and the maximum depth was only 1.2 m, which belonged to shallow failure. The slope height *H* = 10 m, slope ratio = 1⁚1, and infiltration depth *z*_w_ = 1.2 m are selected for the numerical stability analysis of the framework strengthened slope system. The physical and mechanical parameters of soil are listed in Table 1.

**Table 1:** Properties of soil and concrete material.

Range	*γ*_sat_ (kN/m^3^)	*c*' /(kPa)	*φ*' /(°)	*γ*_*c*_ /(kN/m^3^)	*δ*
0 ~ *h*	20	0	45	25	1/3*φ*'
*h* ~ *d*_w_	20	2.4	40		

The frame structure is made of C30 concrete material, and its dimension is based on the design scheme commonly used in engineering, namely *b* = 0.3 m, *h* = 0.3 m, *l*_h_ = 2 m, *l*_v_ = 4 m, and *h*_w_ = 1.4 m. In the numerical analysis, the seepage is not considered. When the seepage is not considered, the permeability force *J* = 0 in Eqs. (7) and (8), and the buoyant unit weight *γ*' is replaced by the saturated unit weight *γ*_sat_. The safety factor for the shallow stability of the slope without protection can then be calculated as *F*_s_ = 1.079 based on Eq. (7). Meanwhile, the safety factor for the shallow slope stability with frame protection can be determined as *F*_s_ = 1.588 based on Eq. (8).

### Numerical analysis and validation

**Figure 5:**
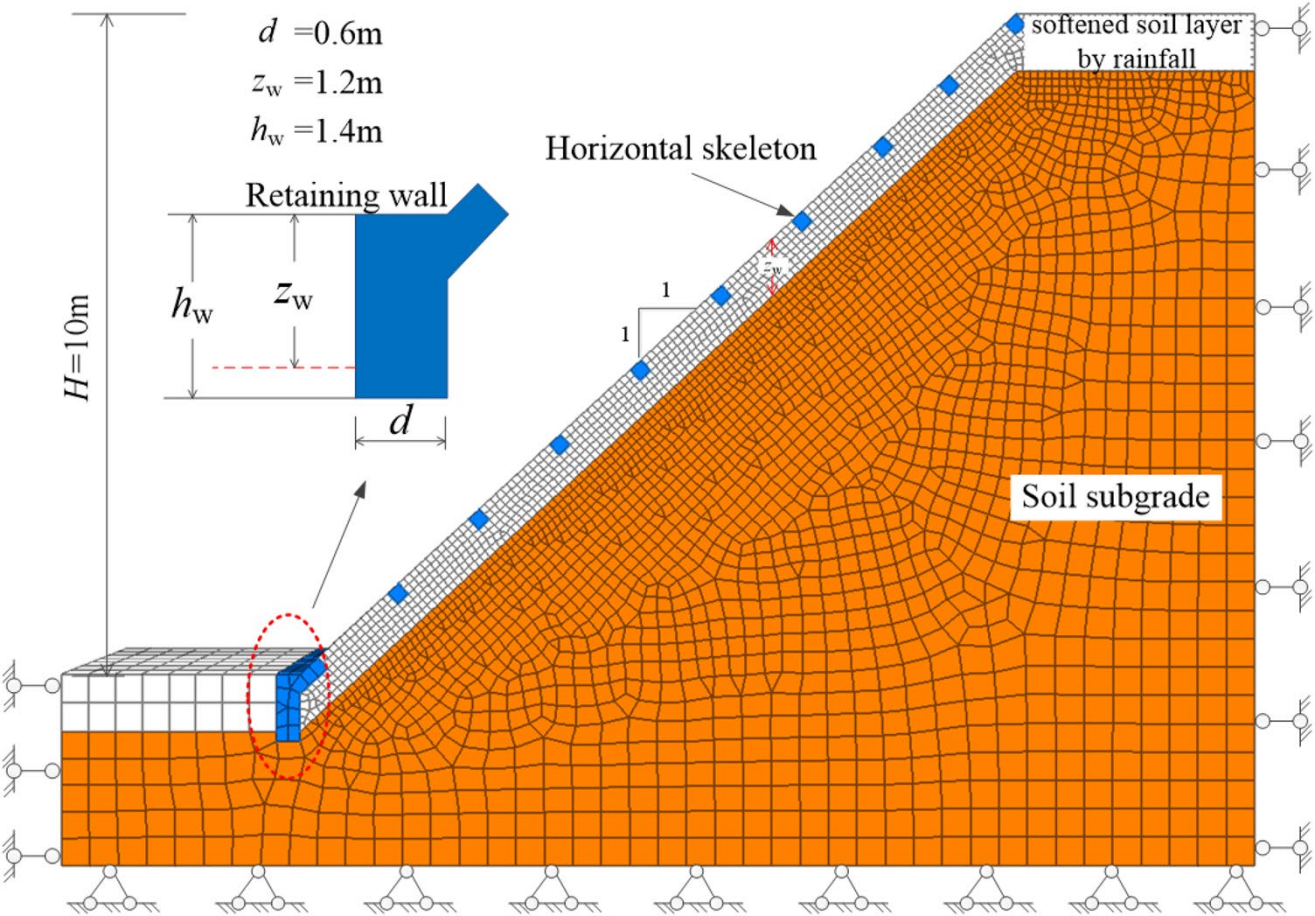
Plan view of the numerical model.

**Figure 6:**
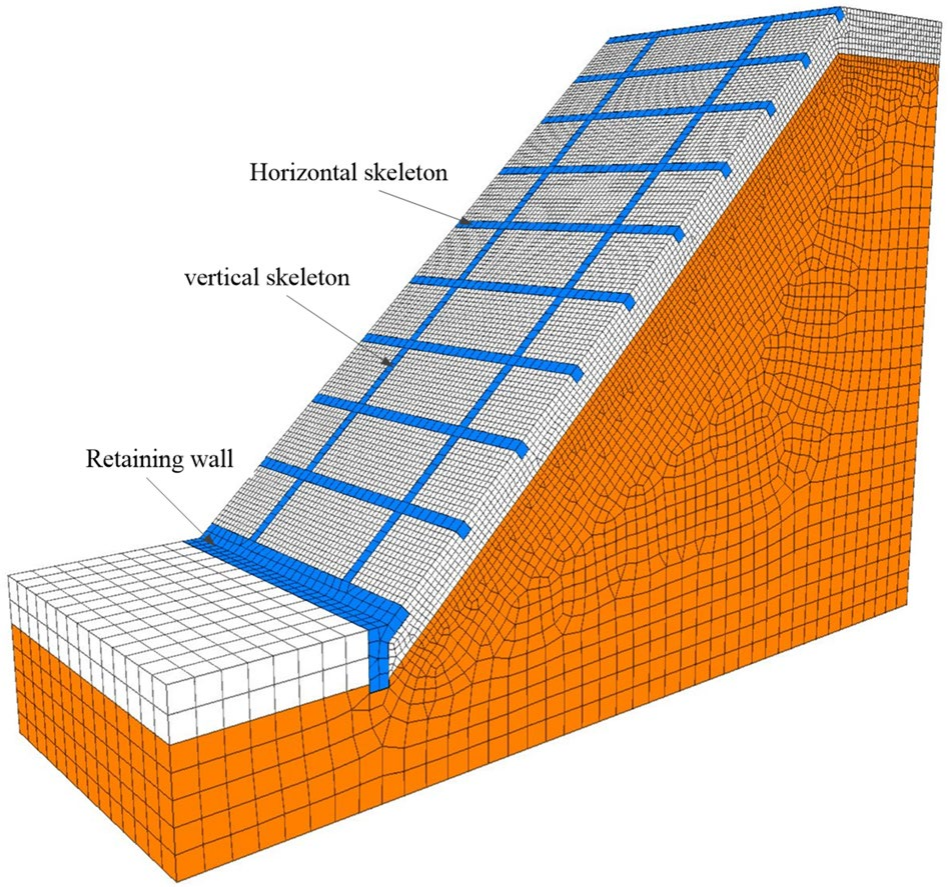
3D view of the numerical model.

The numerical calculation adopts the commercial software of FLAC3D 5.00 based on the finite difference method (FDM). According to the case history and input parameter described above, the numerical model is simplified and established based on symmetry, as shown in Figs. 5 and 6. To avoid boundary effects, the framework model consists of two columns of vertical skeletons. The total width of the model is 2(*l*_h_ + b), as shown in Fig. 6. In the numerical calculation, the frame structure and soil obey elastic and Mohr–Coulomb criteria, and an interface is set between the framework and the soil. The input parameters of soil and concrete material are given in Table 2. The determination of interfacial coefficient refers Wu et al.^19^ as: normal stiffness *k*_n_ = 1.11GN/m^3^, shear stiffness *k*_s_ = 3.7MN/m^3^ for layer 1; normal stiffness *k*_n_ = 2.5 GN/m^3^, shear stiffness *k*_s_ = 11.5 MN/m^3^ for layer 2; the interfacial friction angle and cohesion is taken as 0.8 times of the soil. In fact, the cohesion, internal friction angle, and modulus of the slope soil under rainfall infiltration will continue to change as the saturation of the shallow soil increases. However, the analysis in this paper is focused on investigating the influence of the skeleton protection structure on the stability of the slope under the most unfavorable state (when the shallow soil of the slope reaches saturation), and the geometric parameters of the frame structure on the stability of the shallow slope and material dosage.

**Table 2:** Property of soil and concrete material.

Items	Unit weight *γ* (kN/m^3^)	Young's modulus *E* (MPa)	Posson's ration *υ*	Cohesion *c*′ (kPa)	Friction angle *φ*′ /(°)
Layer1	20	10	0.32	2.4	40
Layer2	21	30	0.30	60	35
Frame structure	23	30,000	0.25		

**Figure 7:**
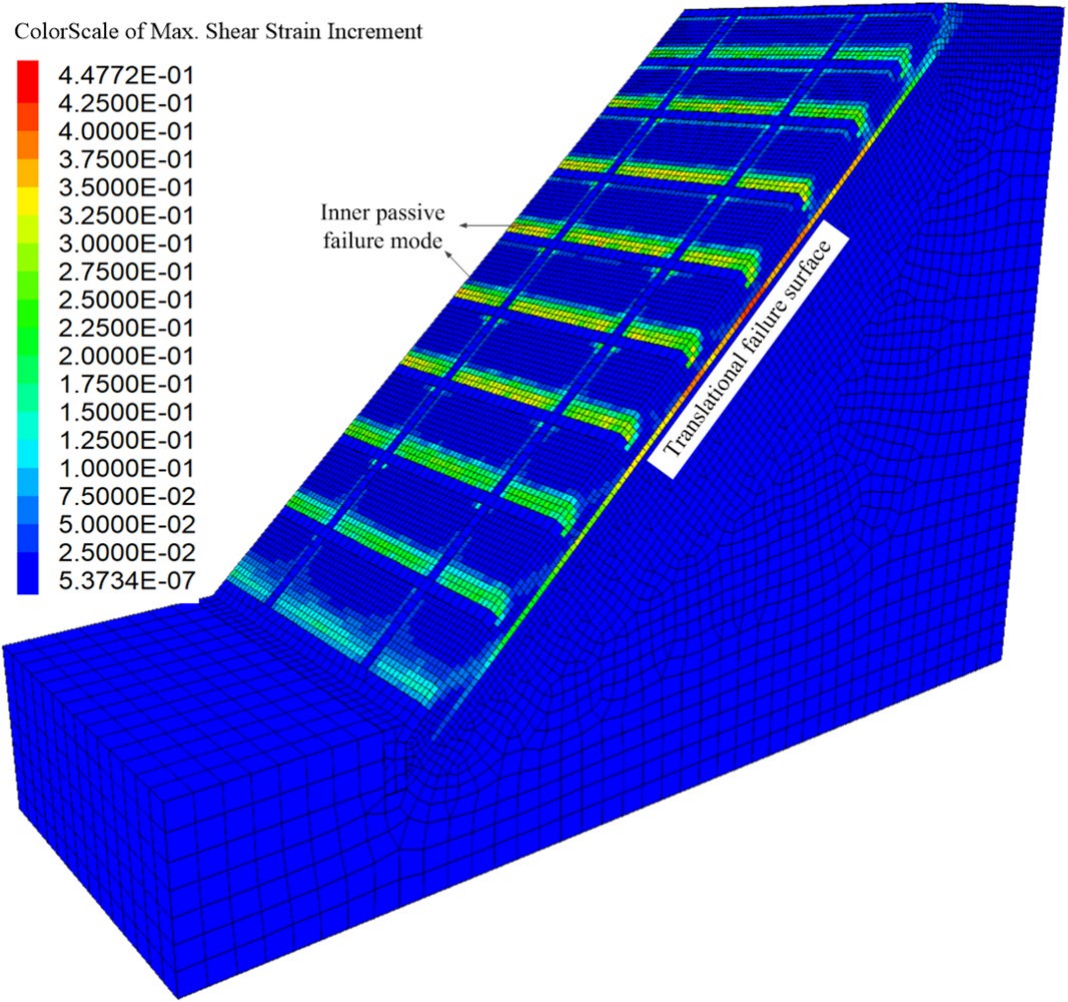
3D view of the maximum shear increment.

**Figure 8:**
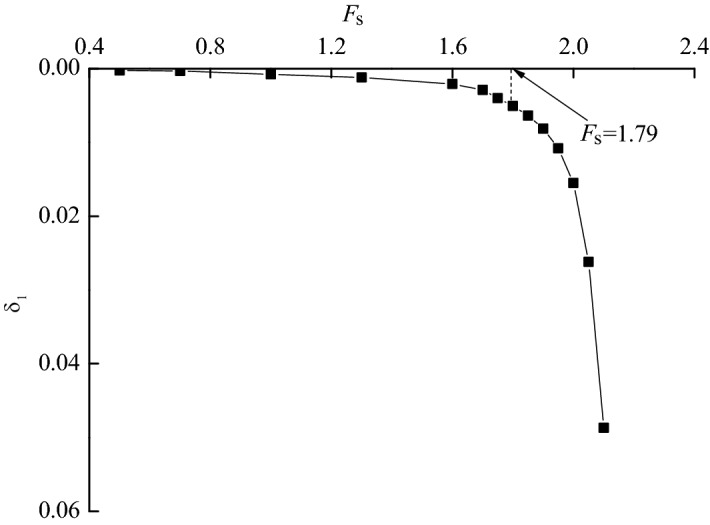
Relationship between *F*_*s*_ and *δ*_1_.

Figures 7 show the three-dimensional contour plot of the maximum shear strain increment when the slope failure occurs. It can be seen that the maximum shear strain increment area appears at the position of the triangular wedge above the transverse framework and the position of the depth of *z*_w_ at the bottom of the shallow soil, which is basically consistent with the failure mode caused by passive earth pressure in Fig. 4. To verify the reasonability of the analytical method, the safety factor of the soil slope is calculated. Figure 8 shows the safety factor of the numerical model is obtained as *F*_*s*_ = 1.79 by taking the displacement mutation as the instability criterion^20^, which is slightly higher than 1.59 calculated by the analytical method. In total, the assumed failure mode (Fig. 4) and computed safety factor agree well with the numerical analysis result, which indicates the analytical method proposed in this paper is reliable.

**Figure 9:**
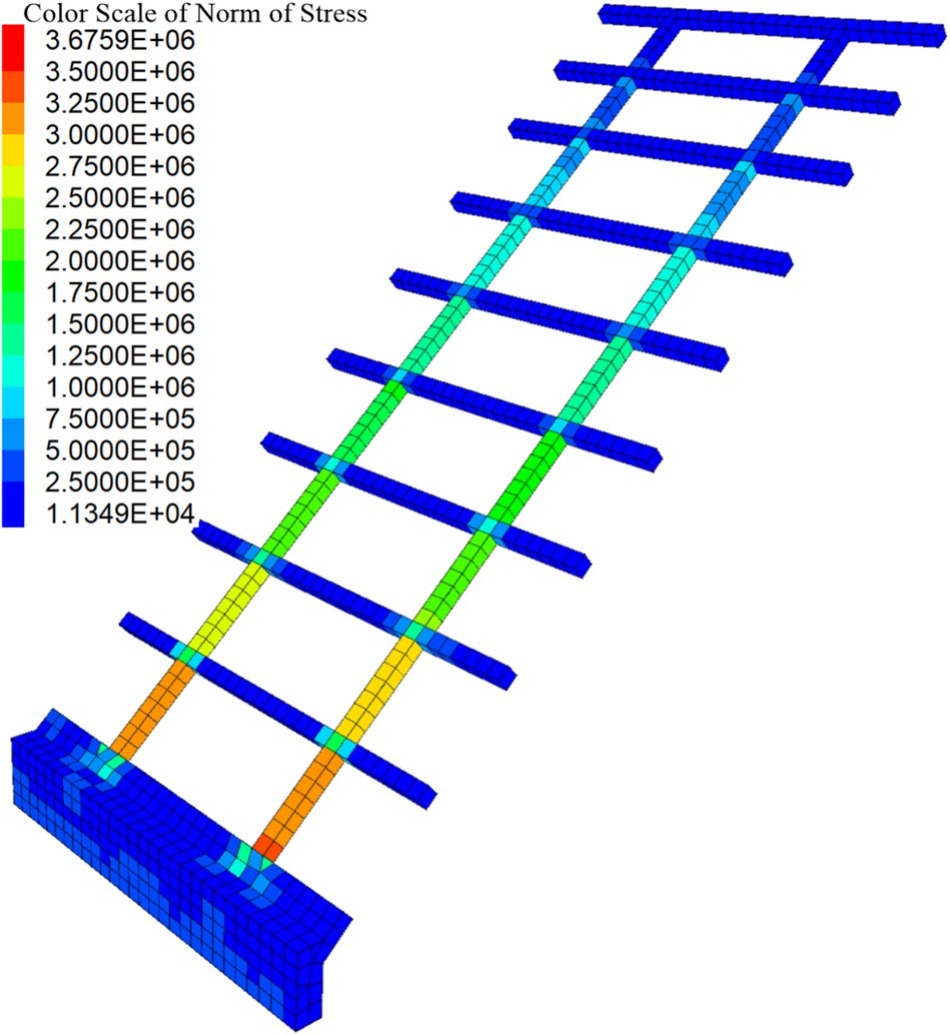
Contour plot of the normal stress for the frame structure(unit: Pa).

**Figure 10:**
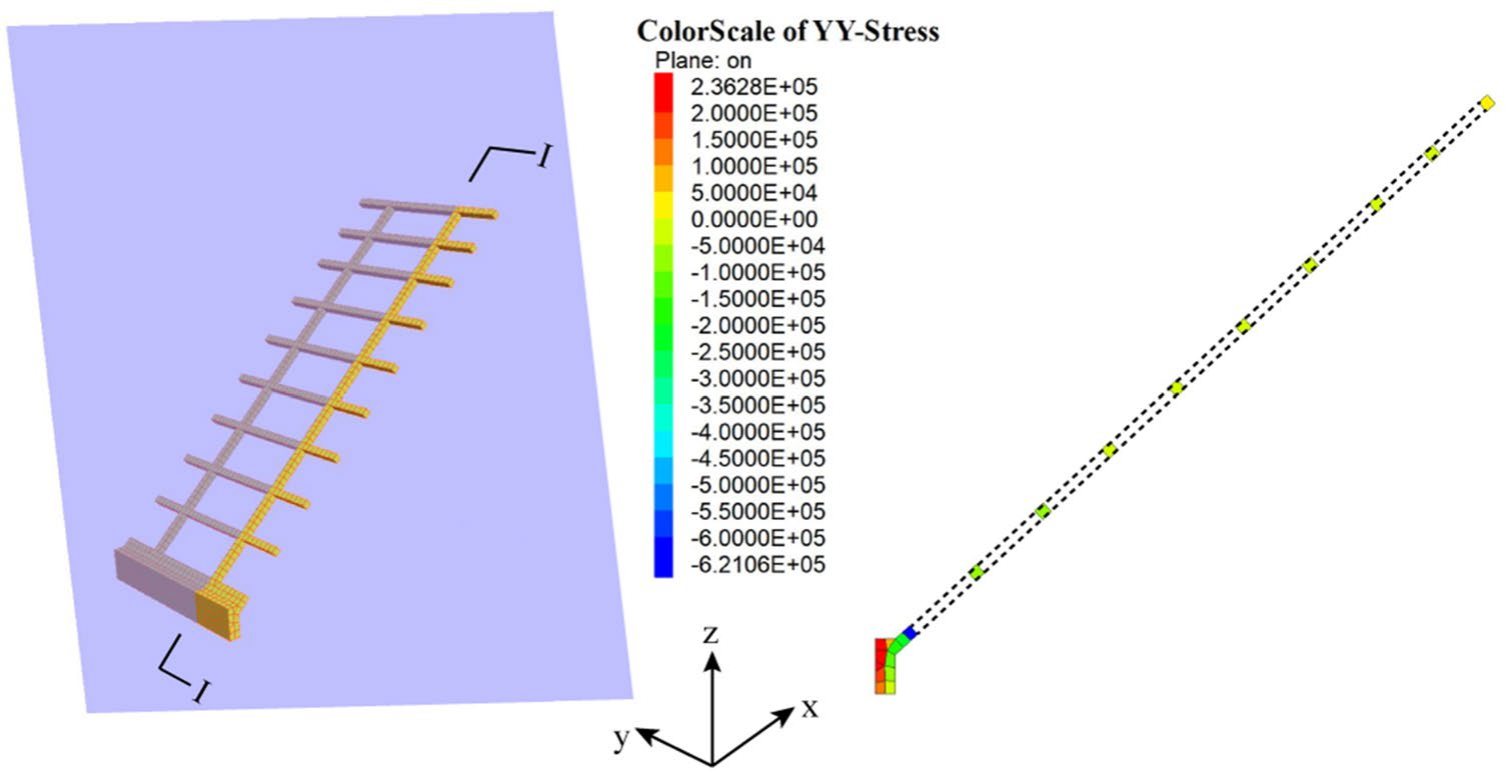
Contour plot of stress acting on the I–I section of the frame structure(unit: Pa).

At the same time, it can be seen that the frame structure is stable as a whole and plays a positive role in protection and reinforcement. Figures 9 and 10 illustrate the contour plots of the structure's normal stress and stress acting on the I–I section when the shallow slope is unstable. It can be seen that the maximum compressive stress of the frame structure is approximately 3.6 × 10^6^ Pa, and the maximum tensile stress is about 6.2 × 10^5^ Pa, which is far less than the strength of C30 concrete (compressive strength: 30 MPa, tensile strength: about 3 MPa). It can be inferred that the frame structure is safe and can serve as an effective measure of protection and reinforcement.

### Influence of structure parameters on the safety factor of shallow slope stability

**Figure 11:**
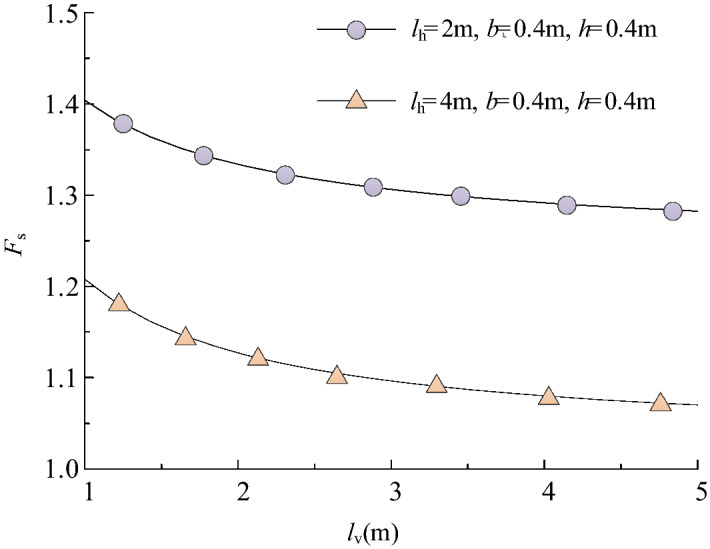
*F*_s_ change with *l*_v_ (slope ratio = 1.5, z_w_ = 1.2 m).

In practical construction, geometric parameters are designed based on experience^21^. Typically, the clear distances *l*_v_ and *l*_h_ are set between 2 and 4 m, and skeleton section width *b* and thickness *h* are 0.3–0.5 m^9,10^. The safety factor *F*_s_ under different vertical clearance *l*_v_ can be derived using Eq. (8), as shown in Fig. 11. It can be seen that the safety factor decreases nonlinearly as *l*_v_ increases, and the larger the horizontal clearance *l*_h_, the smaller the safety factor. In other words, the sparser the frame structure layout, the weaker the protection effect.

**Figure 12:**
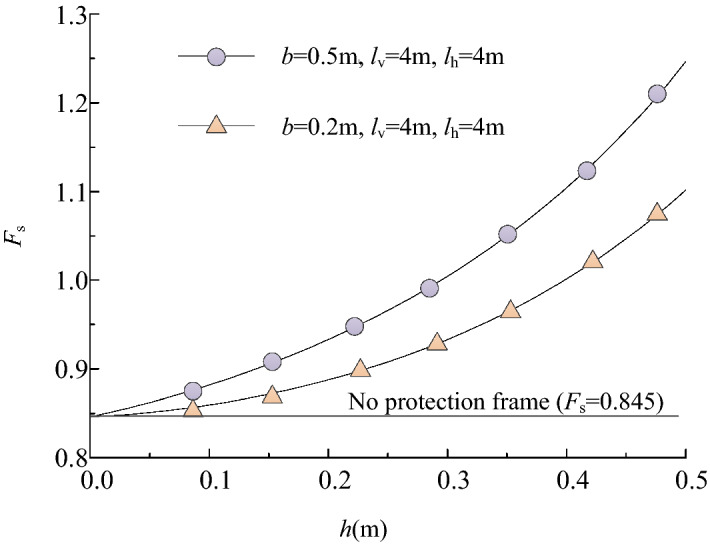
*F*_s_ change with *h* (slope ratio = 1.5, z_w_ = 1.2 m).

Figure 12 shows the changing trend of the safety factor *F*_s_ with the embedded depth *h* and the skeleton width *b*. It can be seen that the safety factor *F*_s_ increases nonlinearly with the increase of the embedded depth *h*, and the larger the frame width *b*, the higher the safety factor. It turns out to be that the increase in the skeleton section's size helps to improve the stability of the shallow slope.

### Parameters sensitivity analysis and optimal scheme

Based on the analysis above, there are four factors that affect the shallow stability of soil slope strengthened by the frame protection structure, i.e., *l*_v_, *l*_h_, *b*, and *h*. To simplify the analysis, the interaction between the factors is not considered. According to the empirical design approach, the sash clearance *l*_v_ and *l*_h_ are 2–4 m, and the skeleton section width *b* and thickness *h* are 0.3–0.5 m^9,10^. At this point, values of each factor level are listed in Table 3.

**Table 3:** Values of the frame structure parameters.

Factor level	*l*_v_/(m)	*l*_h_/(m)	*b*/(m)	*h*/(m)
1	2	2	0.3	0.3
2	3	3	0.4	0.4
3	4	4	0.5	0.5

The calculation involves four factors and three levels. Orthogonal table L_9_ (3^4^) can be selected for calculation; that is, nine test schemes are carried out. At the same time, consider the safety factor *F*_s_ and the concrete amount per unit protection area *V*_A_ as the inspection index, where *V*_A_ is expressed by Eq. (13). The calculation results can be listed in Table 4.

13 $$V_{A} = \frac{{bh(l_{{\text{v}}} + l_{{\text{h}}} + b)}}{{{(}l_{{\text{v}}} {\text{ + b)(}}l_{{\text{h}}} {\text{ + b)}}}}$$

**Table 4:** Orthogonal test schemes and results.

Scheme	*l*_v_	*l*_h_	*b*	*h*	*F*_s_				*V*_A_ (m^3^/m^2^)
					Slope ratio = 1⁚1.5		Slope ratio = 1⁚2.0		
					*z*_w_ = 1.0 m	*z*_w_ = 1.2 m	*z*_w_ = 1.0 m	*z*_w_ = 1.2 m	
1	1	1	1	1	1.205	1.084	1.580	1.422	0.073
2	1	2	2	2	1.510	1.292	2.040	1.731	0.106
3	1	3	3	3	2.150	1.670	3.097	2.325	0.144
4	2	1	2	3	1.658	1.387	2.254	1.865	0.132
5	2	2	3	1	1.150	1.027	1.494	1.361	0.067
6	2	3	1	2	1.244	1.111	1.648	1.469	0.062
7	3	1	3	2	1.293	1.148	1.689	1.500	0.116
8	3	2	1	3	1.349	1.185	1.804	1.576	0.078
9	3	3	2	1	1.066	0.983	1.381	1.277	0.052

According to the calculation results of the safety factor, *F*_s_, and the concrete amount per unit protection area, *V*_A_, in Table 4, including four conditions with different slope ratios and z_w_, Eqs. (12) and (13) can carry out the range analysis of the statistical results, as listed in Table 5. It can be seen that the range *R* corresponding to the embedded depth *h* is the largest; that is, *h* has the most significant impact on the shallow stability of the slope, and the rest are *l*_v_, *b*, and *l*_h_ in order. At the same time, *h* is also the most sensitive factor to the concrete amount under the unit protection area, and the rest are *b*, *l*_v_, and *l*_h_ in order.

**Table 5:** Primary and secondary relations of geometric parameters.

Items	Scheme	*l*_v_	*l*_h_	*b*	*H*
*F*_s_ (slope ratio = 1⁚1.5, *z*_w_ = 1.0 m)	*K*_1_	4.865	4.155	3.797	3.421
	*K*_2_	4.052	4.010	4.234	4.047
	*K*_3_	3.708	4.459	4.593	5.156
	*R*	1.156	0.450	0.796	1.735
	Relations	*h* > *l*_v_ > *b* > *l*_h_			
*F*_s_ (slope ratio = 1⁚2.0, *z*_w_ = 1.0 m)	*K*_1_	6.717	5.523	5.032	4.456
	*K*_2_	5.396	5.338	5.675	5.377
	*K*_3_	4.874	6.126	6.280	7.154
	*R*	1.843	0.788	1.248	2.699
	Relations	*h* > *l*_v_ > *b* > *l*_h_			
*F*_s_ (slope ratio = 1⁚1.5, *z*_w_ = 1.2 m)	*K*_1_	4.046	3.619	3.38	3.094
	*K*_2_	3.525	3.504	3.662	3.551
	*K*_3_	3.316	3.764	3.845	4.242
	*R*	0.730	0.260	0.465	1.148
	Relations	*h* > *l*_v_ > *b* > *l*_h_			
*F*_s_ (slope ratio = 1⁚2.0, *z*_w_ = 1.2 m)	*K*_1_	5.479	4.787	4.468	4.060
	*K*_2_	4.695	4.668	4.873	4.700
	*K*_3_	4.353	5.071	5.186	5.766
	*R*	1.126	0.403	0.718	1.706
	Relations	*h* > *l*_v_ > *b* > *l*_h_			
*V*_A_	*K*_1_	0.323	0.321	0.213	0.192
	*K*_2_	0.261	0.251	0.29	0.284
	*K*_3_	0.246	0.258	0.327	0.354
	*R*	0.077	0.07	0.114	0.162
	Relations	*h* > *b* > *l*_v_ > *l*_h_			

**Figure 13:**
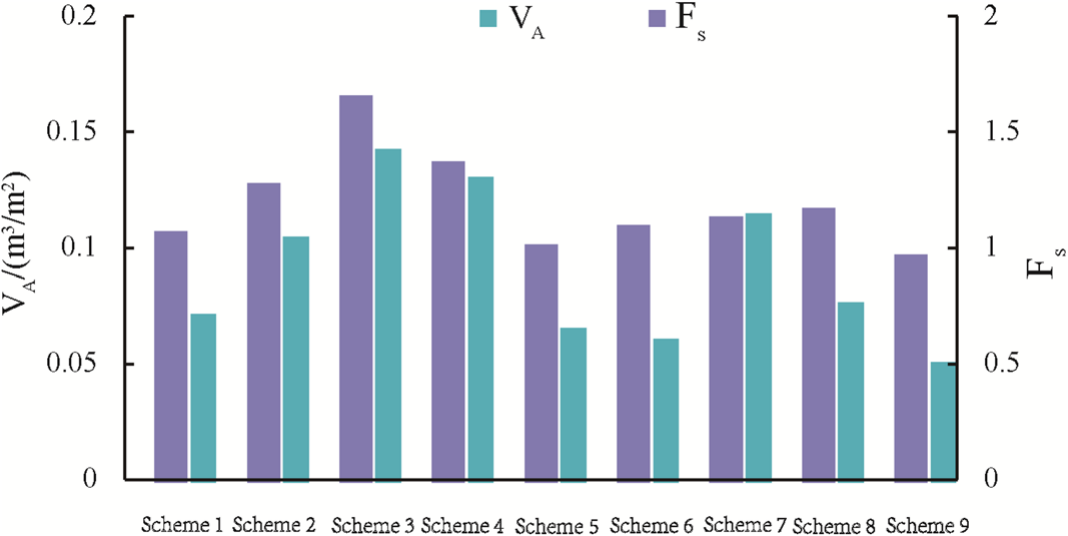
*F*_s_ and V_A_ under different test schemes (slope ratio = 1.5, z_w_ = 1.2 m).

For practical engineering of subgrade slope, it is necessary to meet the safety factor requirement (not less than 1.15 according to^22^) and minimize the material consumption. Figure 13 illustrates the values of *F*_s_ and V_A_ under different test schemes, i.e., nine test schemes as listed in Table 3. Among them, scheme 3 has the highest safety factor, which is the most beneficial to stability. Meanwhile, scheme 3 has the largest value of V_A_, which takes the most considerable amount of construction materials.

In order to balance the relationship between the safety factor and the construction materials, a function, *FV*, is established by standardized the safety factor and the construction materials separately as:

14 $$FV = F_{{{\text{se}}}} - V_{Ae}$$

where $$F_{{{\text{se}}}} = \frac{{F_{{\text{s}}} - F_{{{\text{s}}\min }} }}{{F_{{{\text{s}}\max }} - F_{{{\text{s}}\min }} }}$$ and $$V_{{{\text{Ae}}}} = \frac{V_{{\text{A}}} - V_{\text{A}_{\text{min}}}} {V_{\text{A}_{\text{max}} } - V_{\text{A}_{\text{min}} }}$$.

**Figure 14:**
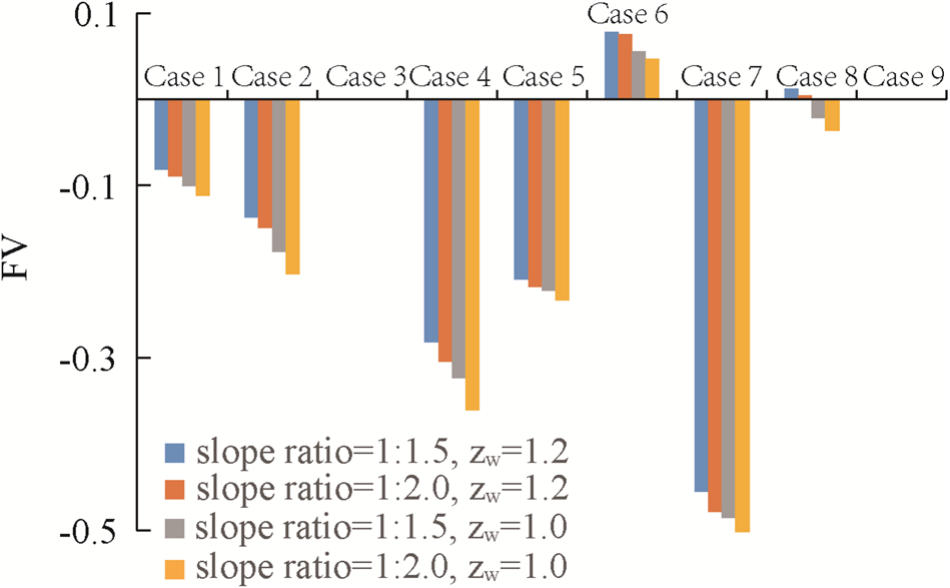
Comparison of *FV* under different schemes.

It can be seen from Eq. (14) that *FV* is the evaluation function of the optimal scheme. When its value is the largest, the scheme is optimal. For this reason, the results are listed in Table 6. Meanwhile, the *FV* results of each scheme are shown in Fig. 14. It can be seen that scheme 6, i.e., (*l*_v_, *l*_h_, *b*, *h*) = (3, 4, 0.3, 0.4 m), with the largest *FV* is the optimal solution, and scheme 7 with the minimal *FV* is the worst solution.

**Table 6:** Calculation of *FV.*

Scheme	*FV*			
	Slope ratio = 1⁚1.5		Slope ratio = 1⁚2.0	
	*z*_w_ = 1.0 m	*z*_w_ = 1.2 m	*z*_w_ = 1.0 m	*z*_w_ = 1.2 m
1	−0.100	−0.081	−0.112	−0.090
2	−0.177	−0.137	−0.203	−0.150
3	0.000	0.000	0.000	0.000
4	−0.324	−0.282	−0.361	−0.305
5	−0.222	−0.209	−0.234	−0.218
6	0.055	0.078	0.047	0.075
7	−0.486	−0.455	−0.516	−0.479
8	−0.022	0.011	−0.036	0.004
9	0.000	0.000	0.000	0.000

## Conclusions

(1) The shallow stability analysis model of soil slope under the protection of the rectangular frame is established. It can be found that the larger the skeleton spacing, the smaller the safety factor of shallow slope stability; the larger the cross-sectional size of the skeleton, the greater the safety factor of shallow slope stability. Therefore, it is feasible to improve the shallow slope stability by reducing the skeleton spacing and increasing the cross-sectional size of the frame structure.

(2) Based on the orthogonal experimental design method, the frame protection structure's parameter sensitivity analysis is carried out. The results show that for the shallow stability of soil slope, the sensitivity factors from large to small are *h*, *l*_v_, *b*, *l*_h_; for the amount of material consumption, the sensitivity factors from large to small are *h*, *b*, *l*_v_, *l*_h_.

(3) To balance the relationship between the safety factor and the construction materials, an optimal scheme evaluation function was constructed. The optimal scheme to the geometric parameters of frame structure commonly used in engineering design is recommended, i.e., (*l*_v_, *l*_h_, *b*, *h*) = (3, 4, 0.3, 0.4 m), which can meet the safety factor required by the specification, and its material consumption is the smallest. Nevertheless, the optimal scheme will also be affected by the physical and mechanical parameters of the slope soil, which should be determined in conjunction with experiments.

## Data Availability

The data are available and explained in this article. Readers can access the data supporting the conclusions of this study.
